# Assessing the Impact of Early Childhood Caries on the Development of First Permanent Molar Decays

**DOI:** 10.3389/fpubh.2019.00186

**Published:** 2019-07-09

**Authors:** Fatma Songur, Sera Simsek Derelioglu, Sinan Yilmaz, Zahide Koşan

**Affiliations:** ^1^Department of Pediatric Dentistry, Faculty of Dentistry, Atatürk University, Erzurum, Turkey; ^2^Department of Public Health, Faculty of Medicine, Atatürk University, Erzurum, Turkey

**Keywords:** early childhood caries, first permanent molar teeth caries, window of infectivity, general anesthesia, ICDAS

## Abstract

**Aim:** The aim of this study was to evaluate whether the treated and untreated severe early childhood caries (ECC) in children would make any impact on the Permanent First Molar (PFM) decays.

**Materials and Methods:** Our descriptive epidemiological study was conducted in Ataturk University, Faculty of Dentistry, Department of Pediatric Dentistry/Erzurum-Turkey, between 2011 and 2017. We included a total of 90 children (44 girls, 46 boys), and divided them into 3 equal groups. They were 6–9 years old with a mean age of 7.38 ± 0.89. Group 1 consisted of the patients who had previously been treated under GA, Group 2 included the patients with untreated ECC and with no previous dental treatment, and Group 3 consisted of the patients who had been periodically treated in normal clinical settings. Each patient was processed through; dmft scoring and PFM caries evaluation process in accordance with International Caries Detection and Assessment System (ICDAS), respectively. The obtained data was analyzed with SPSS v20.0. And also, we used *One-way ANOVA, Kruskal-Wallis and Mann Whitney U* tests.

**Results:** In accordance with ICDAS, we found that Group 3 had the highest mean number of PFMs (2 ± 1.43) and Group 2 had the lowest (1.43 ± 1.45). In Group 2, the number of ICDAS-determined carious PFMs were significantly lower than the other groups (*p* < 0.05). However, ICDAS score 6 was 0 in Group 1, while it was determined higher as 4.2% in Group 2.

**Conclusion:** In respect to our research outcomes, which revealed that regardless of treated or not severe ECC had a significant impact on the PFMs, we strongly recommend that the parent of the children experiencing ECC should be informed about the risk of future caries in PFMs.

## Introduction

Permanent First Molars (PFMs), which usually erupt between ages of 6–7 years are the most critical teeth in the dental arc. Nonetheless, this renders them to be at the highest risk for carious lesions. Hi-carb diets, poor oral hygiene, parental lack of knowledge on the PFM eruption time and previous caries experience are among the predisposing factors for the dental caries in these teeth (1, 2).

It is well-established that caries status of the young permanent teeth is correlated with ECC in the primary dentition (3). However, in predicting the permanent teeth caries, determination of ECC experience is not sufficient alone. Caries presence in the permanent teeth is correlated with the cariogenic factors as well as the caries presence in the primary teeth (4, 5).

Although some researches have already been conducted to determine the impact of ECC that was experienced in the primary dentition, on the permanent teeth caries (6–11), the longitudinal studies should be augmented. Since the studies conducted on this issue were mostly retrospective and data have been collected from the old records, credibility of these researches was limited. In contrast to the retrospective studies, prospective researches are more credible since they utilize the identical and standardized diagnosing methods. However, their biggest disadvantage is the prolonged follow-up time, which may lead to participating patient attrition and loss to follow-ups (12). Thus, the impact of primary tooth caries on dental decays in the permanent dentition is evaluated rather retrospectively (8, 11).

The correlation between the previous caries history and caries development in the permanent teeth has been reported by several researches (3, 7, 13). Gray et al. (14), remarked that history of three or more primary molar caries might be the best predictors for PFM caries development at the age of 7. Similarly, in another research conducted among the Chinese children, authors found a strong correlation between the primary and permanent teeth caries and they emphasized the importance of assessing the caries status in predicting the future risks of caries and also they highlighted the importance of implementing the preventive programs (15).

In their study examining the Mexican children, Vallejos-Sanchez et al. (10) revealed that presence of caries in the primary and permanent teeth was a good predictor for determining the increment of subsequent caries and they also remarked that the previous caries experiences were important in implementing the preventive dental programs. In parallel with all these data given, previous deciduous tooth caries experience was regarded as the main risk factor for future caries development and this indicator was accepted as a “caries predictor” in longitudinal studies (11).

In their study, Tagliaferro et al. (16) re-examined the 13–16 years old children whose primary tooth caries had been evaluated 7 years ago and they concluded that caries in the primary teeth well indicate the caries development in the permanent teeth. They also reported that the risk of new caries development in the children with primary tooth decays during a 7 years period was 2.3 times higher, and they remarked that caries-free status was a protective factor against the future caries development.

In another study, Skeie et al. (17) reported that caries presence on more than two surfaces of the primary second molars was a convenient predicting tool for the future caries risks.

Opposing to all these studies, in a research examining 185 Brazilian children, authors stated that there was no positive relationship between ECC and caries development in the permanent teeth and ECC alone was not a good PFM caries indicator (8).

Early caries development in PFMs of the children in Turkey made us consider reviewing the ECC-PFM caries correlation. A review on caries prediction (5) reporting “*the presence of multiple cavities that will significantly increase the bacterial level in the oral cavity during the second window of infectivity, which also covers especially the PFM eruption period, may affect the development of PFM caries*” was another factor motivated us to conduct this research.

In our study, we aim to assess the future caries development in PFMs of the child patients whose oral health status had been improved through the previous dental treatments in normal clinical settings or under GA and compare their PFM caries to those of previously untreated patients with multiple dental cavities.

The null hypotheses tested were as follows: (1) There was no significant difference between the caries prevalence of PFMs in the children with previously improved oral health but still experiencing ECC and the PFM caries prevalence of the untreated patients also suffering from ECC. (2) Whether treated or not, the children with past ECC experience had higher risks of developing future PFM caries.

## Materials and Methods

### Ethics Committee's Approval and Other Consents

This study was conducted by the Department of Pediatric Dentistry, Faculty of Dentistry, Ataturk University in regard to the provisions of Turkish Ministry of Health Clinical Researches Regulation No.28030 dated as August 19, 2011 and also in accordance with the Faculty of Dentistry Research Ethics Committee's written approval (session No.08/2017 resolution # 53). All participating parents were briefed and informed consent forms were obtained in accordance with Declaration of Helsinki.

### Study Type, Study Time and Place

Our study is a descriptive epidemiological research. The study was conducted in Ataturk University, Faculty of Dentistry, Pediatric Dentistry Department/Erzurum-Turkey between 2011 and 2017. After obtaining all the necessary consents, we retrospectively recruited the children in to the study based on 2011–2015 data. We used convenience sampling method in our study, by choosing the patients from General Anesthesia (GA) medical records. In order for comparing PFM caries, we established a control group by randomly selecting among the child-patients with similar age periods, similar dmft scores and with previous ECC experience, who presented to our clinic in 2017.

### Patient Selection Bias, Study Groups and Their Contents

For participating in the research, the children were required to be between 6 and 9 years old and with no systemic disease. Additionally, they should have previous ECC experiences, their PFMs should be retained in mouth and they should have a minimum dmft index score of 5. We determined the dmft score as 5, since the patients who had previously received dental treatment under GA had a dmft score of minimum 5. And the control group was consisted of the patients with a dmft score of 5, similarly.

Our study evaluating the PFMs in a total of 90 children was divided into 3 groups, each included; 30 patients between the ages of 6 and 9 (at the beginning of the research) with severe ECC experience and who were previously treated under GA in our clinic between 2011 and 2015, 30 patients at the same age range who were normally treated in our clinic in 2017 and another 30 child -patients who were previously untreated.

In order to establish the study groups, we scanned the dental records of 152 patients who had been treated under GA between 2011 and 2015. Seventy six of those patients were excluded from the study since they didn't meet the research criteria. Remaining 76 patients were called by phone but 30 of them didn't participate since they couldn't be reached because they switched their numbers or didn't answer the calls.

Sixteen of 46 patients whom we could reach didn't participate in the study, because they moved to another city and they had some difficulties in intercity traveling or they just avoided involving in the research. Remaining 30 patients were included to establish one of the study groups. The other two groups of patients were chosen among the children who admitted to our clinic in 2017 and also matched the research criteria. We formed the groups of thirty children in order to meet the parametric test conditions. Statistical power of the study was calculated as 78% in the *post-hoc* power analysis. The study groups were as follows;

**Group 1:** This group included children from 6 to 9 years old with severe ECC history and who were previously treated under GA before the PFM eruption in our clinic between 2011 and 2015. They had Stainless Steel Crowns (SSCs) placed in their primary first molars and had a minimum dmft score of 5.
**Group 2:** 6–9 years old patients who presented to our clinic in 2017 with ECC experience and prevalent multiple carious primary teeth but with no previous dental treatment constituted this control group. They had a minimum dmft score of 5 and multi-surface dentinal caries in their primary teeth.
**Group 3:** This study group consisted of 6–9 years old patients with ECC history whose primary teeth had previously been treated in clinical settings and had too many filled or missing primary teeth back then. They also had a minimum dmft score of 5 and multi-surface fillings usually in a single tooth.

### Tools and Techniques for Data Collection

Study data were collected with anamnesis and examination forms improvised by the authors. Anamnesis form was developed for obtaining the patient's individual social information and medical history while the examination form was prepared for evaluating the caries and oral hygiene status. Examination form consisted of sections for dmft/dmfs and Simplified Oral Hygiene Index (OHI-S), and ICDAS scoring for PFMs as well. A single dentist (FS) who received a calibration training in accordance with ICDAS Coordinating Committee Manual (lit http://www.icdas.org) evaluated the caries. Evaluation steps were as follows;

- Filling the anamnesis form.
- Oral hygiene scoring in accordance with OHI-S.
- Determining dmft score after polishing.
- ICDAS scoring consequent to the clinical examination of PFMs.

### Study Indices and Scoring

Caries status of the primary teeth was assessed with dmft/dmfs indices. Teeth extracted due to the caries related reasons were categorized in the (M) dental status code. Exfoliated primary teeth were not included in the (M) category. Anterior teeth were considered having 4 surfaces and posterior teeth were accepted to have 5 surfaces in the dmft calculations. Mean dmft and dmfs values were calculated for all groups.

OHI-S was used to assess oral hygiene status of the patients (18).

Caries status of PFMs were scored with ICDAS and we removed plaque layers from the PFMs for detecting the ICDAS scorings. Four PFMs were evaluated and scored in each patient (PFMs were unrestored and unsealed). Thus, we scored 120 PFMs in each group and 360 PFMs in total. Since ICDAS score frequency for one or more cell was <5, Chi-square analysis would not have a Chi-square distribution and so, our test (*p*-value) would not be valid. Thus, we categorized the ICDAS scores, which were equal or lower than 3 as “non-carious” and the scores higher than 3 as “carious.”

ICDAS scoring was performed by employing the definitions for caries scoring shown in Table 1. Sound tooth surfaces showed no evidence of visible caries (no or questionable change in enamel translucency) when cleaned and after air-dried for 5 s (19).

**Table 1:** Classification of the carious status based upon the International Caries Detection and Assessment System (ICDAS).

**Sound tooth surface: Code 0**
There should be no evidence of caries (either no or questionable change in enamel translucency after prolonged air drying (suggested drying time 5 s). Surfaces with developmental defects such as enamel hypoplasias; fluorosis; tooth wear (attrition, abrasion, and erosion), and extrinsic or intrinsic stains will be recorded as sound. The examiner should also score as sound a surface with multiple stained fissures if such a condition is seen in other pits and fissures, a condition which is consistent with non-carious habits (e.g., frequent tea drinking). This table provides a useful guide for differential diagnosis for carious opacities vs. other opacities
**First visual change in enamel: Code 1**
Code 1: Pits and fissures When seen wet there is no evidence of any change in color attributable to carious activity, but after prolonged air drying (approximately 5 s is suggested to adequately dehydrate a carious lesion in enamel) a carious opacity or discoloration (white or brown lesion) is visible that is not consistent with the clinical appearance of sound enamel OR When there is a change of color because of caries which is not consistent with the clinical appearance of sound enamel and is limited to the confines of the pit and fissure area (whether seen wet or dry). The appearance of these carious areas is not consistent with that of stained pits and fissures as defined in code 0 Code 1: Smooth tooth surfaces When seen wet there is no evidence of any change in color attributable to carious activity, but after prolonged air drying a carious opacity (white or brown lesion) is visible that is not consistent with the clinical appearance of sound enamel. This will be seen from the buccal or lingual surface
**Distinct visual change in enamel: Code 2**
The tooth must be viewed wet. When wet there is a (i) carious opacity (white spot lesion) and/or (ii) Brown carious discoloration which is wider than the natural fissure/fossa that is not consistent with the clinical appearance of sound enamel (Note: the lesion must still be visible when dry)
**Localized enamel breakdown because of caries with no visible dentin or underlying shadow: Code 3**
The tooth viewed wet may have a clear carious opacity (white spot lesion) and/or brown carious discoloration which is wider than the natural fissure/fossa that is not consistent with the clinical appearance of sound enamel. Once dried for approximately 5 s there is carious loss of tooth structure at the entrance to, or within, the pit or fissure/fossa. This will be seen visually as evidence of demineralization [opaque (white), brown, or dark Brown walls] at the entrance to or within the fissure or pit, and although the pit or fissure may appear substantially and unnaturally wider than normal, the dentin is NOT visible in the walls or base of the cavity/discontinuity If in doubt, or to confirm the visual assessment, the WHO/CPI/PSR probe can be used *gently across a tooth surface* to confirm the presence of a cavity apparently confined to the enamel. This is achieved by sliding the ball end along the suspect pit or fissure and a limited discontinuity is detected if the ball drops into the surface of the enamel cavity/discontinuity
**Underlying dark shadow from dentin with or without localized enamel breakdown: Code 4**
This lesion appears as a shadow of discolored dentin visible through an apparently intact enamel surface which may or may not show signs of localized breakdown (loss of continuity of the surface that is not showing the dentin). The shadow appearance is often seen more easily when the tooth is wet. The darkened area is an intrinsic shadow which may appear as gray, blue or brown in color. The shadow must clearly represent caries that started on the tooth surface being evaluated. If in the opinion of the examiner, the carious lesion started on an adjacent surface and there no evidence of any caries on the surface being scored then the surface should be coded “0”
**Distinct cavity with visible dentin: Code 5**
Cavitation in opaque or discolored enamel exposing the dentin beneath The tooth viewed wet may have darkening of the dentin visible through the enamel. Once dried for 5 s there is visual evidence of loss of tooth structure at the entrance to or within the pit or fissure—frank cavitation. There is visual evidence of demineralization [opaque (white), brown, or dark brown walls] at the entrance to or within the pit or fissure and in the examiner judgment dentin is exposed The WHO/CPI/PSR probe can be used to confirm the presence of a cavity apparently in dentin. This is achieved by sliding the ball end along the suspect pit or fissure and a dentin cavity is detected if the ball enters the opening of the cavity and in the opinion of the examiner the base is in dentin. (In pits or fissures the thickness of the enamel is between 0.5 and 1.0 mm. Note the deep pulpal dentin should not be probed)
**Extensive distinct cavity with visible dentin: Code 6**
Obvious loss of tooth structure, the cavity is both deep and wide and dentin is clearly visible on the walls and at the base. An extensive cavity involves at least half of a tooth surface or possibly reaching the pulp

### Statistical Analysis

We used *Statistical Package for Social Sciences* (SPSS v20.0) for analyzing the data collected. Numerical data were presented by standard deviations, arithmetic means, minimum and maximum values, and medians while the categorical data were described by numbers and percentages. With histograms and *Kolmogorov-Smirnov* tests, we checked if the data were normally distributed. We used *one-way ANOVA* test for the multiple comparisons of normally distributed numerical data and *Kruskal-Wallis* test for the non-normal distributions. When the results of *Kruskal-Wallis* test were found to be significant, we used *Mann Whitney U with Bonferroni Correction* test for pairwise comparisons. *Mann Whitney U* test was used again for the comparisons of the paired numerical variables, which were not normally distributed and χ^2^-test was used for analyzing the multiple categorical variables. Correlations between the numerical and ordinal variables were measured with *Spearman Correlation* test. Statistically significant results were obtained when *p* < 0.05.

## Results

The distribution age and gender of the patients by the groups are given in Table 2.

**Table 2:** Distribution of gender and age by the groups.

	**Number**	**Gender**		**Age**
		**Girl**	**Boy**	Mean ± SD^(*)^
Group 1	30	12	18	7.70 ± 0.98
Group 2	30	17	13	7.10 ± 0.88
Group 3	30	15	15	7.33 ± 0.71
Total	90	44 (%48.9)	46 (%51.1)	7.38 ± 0.89

* *Standard deviation*.

Assessments of the mean dmft, dmfs, and OHI-S indices are shown in Table 3. We determined a mean dmft index score of 8.35 ± 1.88 between the participants; and among all groups, Group 1 had the highest mean dmft score while Group 2 had the lowest. According to the results of ANOVA test that was performed for comparing dmft ratios, no statistically significant difference was found between the groups, regarding the mean dmft index score (*p* = 0.32). We found a mean dmfs index score of 20.35 ± 7.53 for all participants. The result of Kruskal Wallis test made for comparing the dmfs index variables of the groups was statistically significant. *Mann Whitney U* test with Bonferroni correction was used for performing pairwise comparisons. Test results revealed that, in terms of the mean dmft index scores, there was a statistically significant difference between Group 1 and 2 and also between Group 2 and 3 (*p* < 0.01 for both pairs). In the test results, mean dmfs index scores of Group 1 and 3 were significantly higher than those of Group 2. We determined a mean OHI-S index score of 0.91 ± 0.44 for all participants. With respect to the results of ANOVA test, which was made for comparing OHI-S index ratios, no statistically significant difference was found between the mean OHI-S indices of the groups (*p* = 0.68).

**Table 3:** Mean dmf-t, dmf-s, and OHI-S indices of the groups.

		**Group 1**	**Group 2**	**Group 3**	***p***
Number		30	30	30	
dmf-t	Min–max	5–12	5–12	5–14	0.32
	Mean ± SD	8.73 ± 1.87	8.00 ±1.89	8.33 ±1.88	
dmf-s	Min–max	12–40	7–33	7–35	<0.01
	Mean± SD Median	24.37 ± 6.09 (24)	15.33 ± 6.19 (15)	21.17 ± 7.56 (20.5)	
OHI-S	Min–max	0–2	0–2	0–2	0.68
	Mean ± SD	0.97 ± 0.44	0.88 ± 0.46	0.90 ± 0.41	

The distributions of ICDAS scores for evaluating the presence of caries in PFMs were expressed in numbers/percentages (Table 4).

**Table 4:** Distribution of ICDAS-scored PFMs by the groups.

		**ICDAS scores**							
**Groups**		**Score 0**	**Score 1**	**Score 2**	**Score 3**	**Score 4**	**Score 5**	**Score 6**	**Sum**
Group 1	Number (%)	28 (23.3%)	16 (13.3%)	17 (14.2%)	24 (20%)	21 (17.5%)	14 (11.7%)	0 (0%)	120 (100%)
Group 2	Number (%)	11 (9.2%)	41 (34.2%)	27 (22.5%)	20 (16.7%)	7 (5.8%)	9 (7.5%)	5 (4.2%)	120 (100%)
Group 3	Number (%)	26 (21.7%)	20 (16.7%)	14 (11.7%)	31 (25.8%)	16 (13.3%)	8 (6.7%)	5 (4.2%)	120 (100%)
Total	Number (%)	65 (18.1%)	77 (21.4%)	58 (16.1%)	75 (20.8%)	44 (12.2%)	31 (8.6%)	10 (2.8%)	360 (100%)

We recognized the PFMs with ICDAS Code 3 and above as “carious” and compared the caries status of the groups, respectively (Table 5). Generally, considering the teeth with ICDAS Code 3 and higher as “carious,” decays were detected in 160 teeth out of a total of 360 PFMs (44.4%) and no caries was found in 200 PFMs (55.6%) in our analysis. Among the groups, Group 3 had the highest caries rate (50%) and Group 2 had the lowest (34.2%). Caries rate in Group 1 was 49.2%. According to the statistical analysis results, there was a significant difference between the groups in terms of caries prevalence distribution (χ^2^ *p* = 0.02) and Group 2 showed the lowest caries prevalence. Group 1 and Group 3 had close caries rates.

**Table 5:** Number of ICDAS-determined caries in all PFMs and their distribution by the groups.

		**Caries status of PFMs in accordance with ICDAS**			
**Groups**		**No caries**	**Caries**	**Total**	***P***
Group 1	Number (%)	61 (50.8%)	59 (49.2%)	120 (100%)	0.02
Group 2	Number (%)	79 (65.8%)	41 (34.2%)	120 (100%)	
Group 3	Number (%)	60 (50%)	60 (50%)	120 (100%)	
Total	Number (%)	200 (55.6%)	160 (44.4%)	360 (100%)	

For each group, distribution of the patients by the number of carious PFMs and their group averages are given in Table 6. The mean numbers of caries in PFMs of the patients in the groups have been compared. In accordance with ICDAS, we found that Group 3 had the highest mean number of carious PFMs (2 ± 1.43) and Group 2 had the lowest (1.43 ± 1.45). Numerical values in Group 1 and Group 2 were close and lower in Group 2. We found no statistically significant difference between the groups. The number of patients with all four PFMs decayed were the highest in Group 1. Mean number of carious PFMs was determined as 1.80 ± 1.52 in all children.

**Table 6:** Distribution of the patients by the number of carious PFMs and their group averages.

		**DMFT index for PMSs**					Mean DMFT indices for PFMs Mean ± SD(*)
**Groups**	**Number**	**No caries number** **(%)**	**1 caries number** **(%)**	**2 caries number** **(%)**	**3 caries number** **(%)**	**4 caries number** **(%)**	
Group 1	30	9 (30%)	4 (13.3%)	5 (16.6%)	3 (10%)	9 (30%)	1.97 ± 1.65
Group 2	30	10 (33.3%)	9 (30%)	4 (13.3%)	2 (6.6%)	5 (16.6%)	1.43 ± 1.45
Group 3	30	6 (20%)	6 (20%)	6 (20%)	6 (20%)	6 (20%)	2 ± 1.43
Total	90	25 (27.7%)	19 (21.1%)	15 (16.6%)	11 (12.2%)	20 (22.2%)	1.80 ± 1.52
							Kruskal Wallis *p* = 0.28

* *Standard deviation*.

## Discussion

Only few longitudinal follow-up studies reporting “how past caries experiences may affect the future oral health” have been conducted so far. In our study, we aimed to search the impact of ECC on PFM decays by comparing PFM caries in the patients whose oral health conditions were improved with the dental treatments under GA or with the treatments in normal clinical settings to the caries in the patients with multiple cavities who were not previously treated.

Several researches reported that the caries history in primary dentition was a predisposing factor for the caries in permanent teeth, although their study designs were different from ours (15, 20–24). All of these studies in the literature included only the follow-ups of the patients experienced ECC in the past, they did not include monitoring of the patients with treated ECC. Thus, since our research is the first study in which the caries status of PFMs in patients with previously treated ECC has been evaluated; currently there is no sufficient literature data to be compared with.

In our study, we determined that the caries rate in PFMs of the 6–9 years old children with past ECC experience was 44.4%. Even though no secondary caries presented because some of these children with severe ECC had previously been treated under GA and thus, dental caries in their primary teeth had been managed and then the teeth were restored with SSCs, permanent tooth caries had not been prevented yet. When the number of carious PFMs, which had been determined in accordance with the ICDAS scores of the groups was assessed; Group 2 showed the lowest caries prevalence and there was a significant difference between the groups regarding the prevalence of caries distribution. With this fact, the first null hypothesis stating “*There was no significant difference between the caries prevalence of PFMs in the children with previously improved oral health but still experiencing ECC and the PFM caries prevalence of the untreated patients also suffering from ECC*” was rejected.

Although all groups had similar dmft scores, the reason for obtaining lower PFM caries rates in Group 2, which included the patients with untreated primary teeth carious lesions can be explained by its lower dmfs rates. In Group 2, parents might have not made their children treated, since they had less severe and asymptomatic caries.

Early childhood dental visits provided by most families may indicate the children's early dental problems rather than the parental responsibilities (25–27). In this situation, we may consider that however the dental cavities of the children who experienced primary tooth caries at very young ages had already been removed prior to the eruption of PFMs, problems related with teeth structures, genetic factors, ineffective tooth brushing techniques and insufficient self-training levels of the families might also have increased the caries rate in the permanent teeth (28–30). Moreover, even if the dental cavities had been eliminated, some bacterial causes; such as a certain size of bacterial population remained constant in oral cavity due to the previous bacterial exposure in high levels might also have affected this condition (31). Further studies are needed to assess the bacterial levels. Factors associated with early caries in the primary teeth can also cause the same patient to develop permanent tooth caries.

Our study showed that the removal of the cavities in the primary teeth before the eruption of the permanent teeth had no positive impact in declining the development of the permanent teeth caries. However, we should also keep in mind that the patients treated under GA developed high rates of dmft scores at much earlier ages.

In our study, mean number of carious PFMs was determined as 1.80 ± 1.52 in the children at the maximum age of 9 and with past ECC experience, regardless of they had been previously treated or not. Thus, the second null hypothesis that “*whether treated or not, the children with past ECC experience had higher risks of developing future PFM caries*,” was accepted.

In our study, the rate of carious PFMs was lower in the patient group with untreated permanent tooth decays, who visited our clinic for the first time (Group 2). In contrast to our expectations, number of the carious PFMs were higher in the Group 1, in which ECC were comprehensively treated under GA. According to these results, in this patient population with previous ECC history, presence of untreated primary teeth caries in the permanent teeth eruption period alone is not a strong predictor for caries development in the permanent dentition. Nevertheless, further studies determining the bacterial levels are needed for extensive evaluations.

The primary purpose of our study was to search the impact of treated and untreated ECC in children, on PFM decays. Besides, the reason for including Group 3 in the study was the consideration of establishing a third group involving multi-session treatments so that the caries would be managed over a long period of time, in order for comparing it with Group 1, in which better restorations were placed and caries were removed under GA.

We used SSCs in the posterior restorations placed under GA but compomers were used in the posterior restorations placed in Group 3, because the child-patients were very young. Researches reported that SSC restorations had superior durability and longevity than the class II amalgam and resin-based composite restorations (32).

In the light of above information, we aimed to search how an age-associated long-term ECC treatment that was carried-out in normal clinic environment would affect the PFM development by comparing it to a single-visit treatment, which was performed under GA and with better restorative materials (e.g., SSCs especially in the teeth adjacent to PFMs).

In accordance with the data obtained, we found that removal of dental caries in the children with previous ECC experience alone had not decreased the risk of developing PFM decays. Thus, although the dental treatments have been provided for the patients who experienced carious lesions especially at very young ages, frequent follow-up visits and supervising the dental preventive programs are also important in order for maintaining the dental health during the permanent tooth eruption period. In this issue, the dentists' role includes; informing the parents of the ECC-experiencing children about PFM caries risks, ensuring regular, and effective caries-preventive applications, explaining the importance of oral care and periodic dental check-ups to the parents and encouraging them.

## Conclusion

The data obtained in this study revealed that regardless of having been treated or not, ECC posed a risk for developing PFM caries. However, since we could follow-up only a limited number of child-patients, further clinical studies with extensive patient groups are needed.

## Ethics Statement

This study has been conducted in Department of Pediatric Dentistry-Ataturk University Faculty of Dentistry, Erzurum in accordance with the provisions of Ministry of Health regulation 28030, dated as 19 August 2011 (court # 08/2017, Resolution # 53) and was based on the signed parental informed consents.

## Author Contributions

FS: scanning archive data, control of patients, examination of patients, and article writing. SS: study design, sharing the archive data for this study, article writing, and English translation of the article. SY and ZK: statistically analyses and article writing.

### Conflict of Interest Statement

The authors declare that the research was conducted in the absence of any commercial or financial relationships that could be construed as a potential conflict of interest.

## References

[1] Luca R Stanciu I Ivan A Vinereanu A Knowledge on the first permanent molar-audit on 215 Romanian mothers. OHDMBSC. (2003) 2:27–32.

[2] Zouashkiani T Mirzakhan T Parental knowledge about presence of the first permanent molar and its effect on health of the this tooth in 7–8 years-old children (2006). J Mash Dent Sch. (2006) 30:225–32.

[3] Powell LV. Caries prediction: a review of the literature. Community Dent Oral Epidemiol. (1998) 26:361–71. 10.1111/j.1600-0528.1998.tb01974.x 9870535

[4] Eriksen HM Dimitrov V. Ecology of oral health: a complexity perspective. Eur J Oral Sci. (2003) 111:285–90. 10.1034/j.1600-0722.2003.00053.x 12887392

[5] Powell LV. Caries risk assessment: relevance to the practitioner. JADA. (1998) 129:349–53. 10.14219/jada.archive.1998.0209 9529810

[6] D'Mello GI Long-term Oral and General Health Outcomes in Adolescents who had Extensive Decay in Early Childhood (Thesis, Doctor of Clinical Dentistry). University of Otago (2011).

[7] Raadal M Espelid I. Caries prevalence in primary teeth as a predictor of early fissure caries in permanent first molars. Community Dent Oral Epidemiol. (1992) 20:30–4. 10.1111/j.1600-0528.1992.tb00669.x 1547609

[8] Silva FA Oliveira CAGR Tannure PN de Paula VAC de Souza SouzaIPR Early childhood caries as a possible predictor for caries in first permanent molars. Int J Dev Res. (2014) 4:1085–7.

[9] Topaloglu-Ak A Eden E Caries in primary molars of 6–7-year-old Turkish children as risk indicators for future caries development in permanent molars. J Dent Sci. (2010) 5:150–5. 10.1016/S1991-7902(10)60022-0

[10] Vallejos-Sanchez AA Medina-Solís CE Casanova-Rosado JF Maupomé G Minaya-Sanchez M Pérez-Olivares S. Caries increment in the permanent dentition of Mexican children in relation to prior caries experience on permanent and primary dentitions. J Dent. (2006) 34:709–15. 10.1016/j.jdent.2006.01.003 16494985

[11] Zemaitiene M Grigalauskiene R Andruskeviciene V Matulaitiene ZK Zubiene J Narbutaite J. Dental caries risk indicators in early childhood and their association with caries polarization in adolescence: a cross-sectional study. BMC Oral Health. (2016) 17:2. 10.1186/s12903-016-0234-8 27412383 PMC4942946

[12] Caruana EJ Roman M Hernández-Sánchez J Solli P. Longitudinal studies. J Thorac Dis. (2015) 7:E537–40. 10.3978/j.issn.2072-1439.2015.10.63 26716051 PMC4669300

[13] Honkala E Nyyssönen V Kolmakow S Lammi S. Factors predicting caries risk in children. Eur J Oral Sci. (1984) 92:134–40. 10.1111/j.1600-0722.1984.tb00869.x 6585920

[14] Gray M Marchment M Anderson R. The relationship between caries experience in the deciduous molars at 5 years and in first permanent molars of the same child at 7 years. Community Dent Health. (1991) 8:3–7. 2049653

[15] Li Y Wang W. Predicting caries in permanent teeth from caries in primary teeth: an eight-year cohort study. J Dent Res. (2002) 81:561–6. 10.1177/154405910208100812 12147748

[16] Tagliaferro EP Pereira AC Meneghim Mde C Ambrosano GM. Assessment of dental caries predictors in a seven-year longitudinal study. J Public Health Dent. (2006) 66:169–73. 10.1111/j.1752-7325.2006.tb02575.x 16913242

[17] Skeie M Raadal M Strand G Espelid I. The relationship between caries in the primary dentition at 5 years of age and permanent dentition at 10 years of age–a longitudinal study. Int J Pediatr Dent. (2006) 16:152–60. 10.1111/j.1365-263X.2006.00720.x 16643535

[18] Greene JG Vermillion JR. The simplified oral hygiene index. JADA. (1964) 68:7–13. 10.14219/jada.archive.1964.0034 14076341

[19] Ismail A Sohn W Tellez M Amaya A Sen A Hasson H. The International Caries Detection and Assessment System (ICDAS): an integrated system for measuring dental caries. Community Dent Oral Epidemiol. (2007) 35:170–8. 10.1111/j.1600-0528.2007.00347.x 17518963

[20] Grund K Goddon I Schüler IM Lehmann T Heinrich-Weltzien R. Clinical consequences of untreated dental caries in German 5-and 8-year-olds. BMC Oral Health. (2015) 15:140. 10.1186/s12903-015-0121-8 26538196 PMC4634920

[21] Nunes-Dos-Santos DL Almeida De Deus Moura LDF Deus Moura Lima MD Soares Pereira Lopes T Silva De Moura M Is severe early childhood caries predictive of caries and fluorosis in permanent teeth? Ten-year follow-up. Rev Odontol UNESP. (2017) 46:164–73. 10.1590/1807-2577.17916

[22] Peretz B Ram D Azo E Efrat Y. Preschool caries as an indicator of future caries: a longitudinal study. Pediatr Dent. (2003) 25:114–8. 12723835

[23] van Palenstein Helderman W Van't Hof M Van Loveren C. Prognosis of caries increment with past caries experience variables. Caries Res. (2001) 35:186–92. 10.1159/000047454 11385198

[24] Vanobbergen J Martens L Lesaffre E Bogaerts K Declerck D. The value of a baseline caries risk assessment model in the primary dentition for the prediction of caries incidence in the permanent dentition. Caries Res. (2001) 35:442–50. 10.1159/000047488 11799285

[25] American Academy of Pediatric Dentistry Policy on early childhood caries (ECC): classifications, consequences, and preventive strategies. Pediatr Dent. (2008) 30 (Suppl. 7):40. 19216381

[26] Çolak H Dülgergil ÇT Dalli M Hamidi MM. Early childhood caries update: a review of causes, diagnoses, and treatments. J Nat Sc Biol Med. (2013) 4:29. 10.4103/0976-9668.107257 23633832 PMC3633299

[27] Hussein AS Abu-Hassan MI Schroth RJ Ghanim AM Parent's perception on the importance of their children's first dental visit (A cross-sectional pilot study in Malaysia). JODR. (2013) 23:1–6. 10.12816/0012189

[28] Hajishengallis E Parsaei Y Klein MI Koo H. Advances in the microbial etiology and pathogenesis of early childhood caries. Mol Oral Microbiol. (2017) 32:24–34. 10.1111/omi.12152 26714612 PMC4929038

[29] Leong PM Gussy MG Barrow SYL de Silva-Sanigorski A Waters E. A systematic review of risk factors during first year of life for early childhood caries. Int J Pediatr Dent. (2013) 23:235–50. 10.1111/j.1365-263X.2012.01260.x 22925469

[30] Li Y Ge Y Saxena D Caufield P. Genetic profiling of the oral microbiota associated with severe early-childhood caries. J Clin Microbiol. (2007) 45:81–7. 10.1128/JCM.01622-06 17079495 PMC1828962

[31] Selwitz RH Ismail AI Pitts NB. Dental caries. Lancet. (2007) 369:51–9. 10.1016/S0140-6736(07)60031-2 17208642

[32] Seale NS Randall R. The use of stainless steel crowns: a systematic literature review. Pediatr Dent. (2015) 37:145–60. 25905656

